# Innovative sample preparation using alcohol dehydration and high refractive index medium enables acquisition of two‐channel super‐resolution 3D STED image of an entire oocyte

**DOI:** 10.1111/jmi.13363

**Published:** 2024-10-11

**Authors:** Michaela Frolikova, Michaela Blazikova, Martin Capek, Helena Chmelova, Jan Valecka, Veronika Kolackova, Eliska Valaskova, Martin Gregor, Katerina Komrskova, Ondrej Horvath, Ivan Novotny

**Affiliations:** ^1^ Laboratory of Reproductive Biology Institute of Biotechnology of the Czech Academy of Sciences BIOCEV Prumyslova Vestec Czech Republic; ^2^ Light Microscopy Core Facility Institute of Molecular Genetics of the Czech Academy of Sciences Prague Czech Republic; ^3^ Laboratory of Biomathematics Institute of Physiology of the Czech Academy of Sciences Prague Czech Republic; ^4^ Centre of the Region Hana for Biotechnological and Agricultural Research Institute of Experimental Botany of the Czech Academy of Sciences Olomouc Czech Republic; ^5^ Laboratory of Integrative Biology Institute of Molecular Genetics of the Czech Academy of Sciences Prague Czech Republic; ^6^ Department of Zoology Faculty of Science Charles University Prague Czech Republic

**Keywords:** alcohol dehydration, 3D STED, high refractive index medium, large biological objects, oocyte, sample preparation, super‐resolution

## Abstract

Super‐resolution (SR) microscopy is a cutting‐edge method that can provide detailed structural information with high resolution. However, the thickness of the specimen has been a major limitation for SR methods, and large biological structures have posed a challenge. To overcome this, the key step is to optimise sample preparation to ensure optical homogeneity and clarity, which can enhance the capabilities of SR methods for the acquisition of thicker structures.

Oocytes are the largest cells in the mammalian body and are crucial objects in reproductive biology. They are especially useful for studying membrane proteins. However, oocytes are extremely fragile and sensitive to mechanical manipulation and osmotic shocks, making sample preparation a critical and challenging step.

We present an innovative, simple and sensitive approach to oocyte sample preparation for 3D STED acquisition. This involves alcohol dehydration and mounting into a high refractive index medium. This extended preparation procedure allowed us to successfully obtain a unique two‐channel 3D STED SR image of an entire mouse oocyte. By optimising sample preparation, it is possible to overcome current limitations of SR methods and obtain high‐resolution images of large biological structures, such as oocytes, in order to study fundamental biological processes.

Lay Abstract: Super‐resolution (SR) microscopy is a cutting‐edge tool that allows scientists to view incredibly fine details in biological samples. However, it struggles with larger, thicker specimens, as they need to be optically clear and uniform for the best imaging results. In this study, we refined the sample preparation process to make it more suitable for SR microscopy. Our method includes carefully dehydrating biological samples with alcohol and then transferring them into a mounting medium that enhances optical clarity. This improved protocol enables high‐resolution imaging of thick biological structures, which was previously challenging. By optimizing this preparation method, we hope to expand the use of SR microscopy for studying large biological samples, helping scientists better understand complex biological structures.

## 1 INTRODUCTION

Studying biological structures in high detail is crucial for understanding their roles in various cellular processes. However, traditional optical microscopy is limited by the diffraction barrier, restricting our ability to observe subcellular structures in fine detail. Recently, a variety of super‐resolution (SR) methods have been developed, based on several main approaches that allow us to overcome the diffraction limit barrier.^1^ Among these techniques, Stimulated Emission Depletion (STED) microscopy has become a powerful method for investigating the subcellular structure of biological samples, providing the ability to achieve resolutions of tens of nanometres in all three spatial dimensions. Unlike Structured Illumination Microscopy (SIM) which improves resolution by a factor of two, STED provides significantly higher resolution. On the other hand, while Single Molecule Localisation Microscopy (SMLM) can achieve even higher resolution than STED, STED has the distinct advantage of being able to work effectively with thicker samples.^1^

STED utilises a confocal scanning microscope equipped with an additional doughnut‐shaped depletion laser.^2^, ^3^ The doughnut‐shaped depletion laser is carefully co‐aligned with the excitation laser and, in the overlapping region, stimulated emission depletion of fluorochromes occurs. This phenomenon effectively shrinks the region of sample emission and increases the final lateral resolution of the acquired images.^4^, ^5^ Higher axial resolution may be achieved by using a 3D STED technique when a phase plate is inserted into the depletion laser beam path. This allows the formation of a so‐called z‐doughnut shape over and below excitation PSF and thus enhances the axial resolution of the final image.^6^

Although confocal scanning microscopy is robust, STED microscopy, especially 3D STED, is highly sensitive to the precise co‐alignment of excitation and depletion lasers. Any misalignment can drastically reduce signal intensity and degrade image resolution. This issue is enhanced by spherical aberration, which occurs when the sample's refractive index is incompatible with the objective lens, leading to PSF smearing and further misalignment.^7^

While 3D STED achieves impressive resolution in thin samples, imaging thicker structures is challenging, due to spherical aberration in deeper layers. To correct this, glycerol immersion objectives, with a refractive index closer to biological tissues, can be used. However, these objectives have a lower numerical aperture (NA) than high NA oil objectives, resulting in compromised resolution. In addition, a correction collar is often required to address refractive index mismatch, adding complexity and further limiting resolution compared to high NA oil objectives.^8^

The second approach for correcting spherical aberration is on the sample preparation side. By mounting the sample in a high refractive index (RI) medium, it becomes possible to use a high NA oil objective, thereby maximising resolution. One particularly effective high RI medium is 2,2'‐thiodiethanol (TDE), which is both index‐tunable and compatible with fluorescence imaging. TDE, being miscible with both water and ethanol at any ratio, allows precise adjustment of the sample's RI to match the immersion oil, significantly reducing spherical aberration. This not only preserves image brightness and resolution, but also maintains the fluorescence quantum yield of most fluorophores, making it an ideal medium for deep tissue imaging.^9^ One type of sample that often presents a significant challenge due to pronounced spherical aberration is oocytes. This problem becomes particularly significant when attempting to acquire high‐resolution or even SR images of these large objects, where spherical aberration can severely degrade image quality and resolution.

Oocytes are the largest cells in the mammalian body, with mouse oocytes having an average diameter of 80 µm. They contain a large amount of cytoplasm, which causes high pressure on the oocyte plasma membrane, making them fragile and sensitive to manipulation. Furthermore, their perfectly round shape makes them completely nonadherent, preventing them from adhering to slides. Consequently, during the immunostaining procedure, the oocytes are transferred between various solutions using a glass pipette. The critical part of the immunostaining procedure is the final transfer of the sample from the water‐based staining solution to a high refractive index mounting medium. In adherent cells, a common step before this transfer is air‐drying the sample. This removes water residues and ensures a homogeneous penetration of the imaging medium into the sample, avoiding potential refractive index mismatches and downstream optical aberrations.^10^ Unfortunately, in oocytes, air‐drying leads to the collapse of their fragile structure. Therefore, instead of air‐drying, the oocytes are transferred from the water‐based staining solution to the mounting medium through a series of gradually increasing glycerol concentrations.^11^ However, this procedure does not seem effective enough to remove all water residues from the oocyte and its surroundings and does not solve the spherical aberration while the oil objective is used in combination with mounting media with relatively lower RI. This results in a significant deterioration in the quality of microscopic images obtained from the deeper parts of the sample.

In some protocols, residual water is removed by dehydration through a series of solutions with increasing alcohol concentrations, a technique well‐established in sample preparation of germ cells (including oocytes) for electron microscopy^12^, ^13^ and fluorescence in situ hybridisation.^14^ However, this alcohol dehydration method is less common in standard immunostaining procedures for light microscopy, despite its ability to avoid structural deformations of the sample.

In this paper, we present the successful implementation of novel sample preparation steps that combine gentle dehydration with a gradual transition to a TDE‐based mounting medium with a refractive index equal to glass. This sample adjustment procedure significantly reduced spherical aberration, allowing the use of a high NA oil objective. The enhanced optical quality of the sample enabled 3D STED imaging at depths within the sample that have never before been achieved for an entire oocyte. We demonstrate a full 3D STED image that spans the entire depth of the sample.

Although this study focuses on oocytes, the optimised sample preparation method developed here has broad potential for improving super‐resolution imaging across a wide range of biological specimens, enabling high‐resolution imaging of large and more complex structures.

## 2 MATERIAL AND METHODS

### 2.1 Animals

Inbred C57BL/6J female mice were housed in a breeding colony of the Laboratory of Reproduction, IMG animal facilities, Institute of Molecular Genetics of the Czech Academy of Sciences. Food and water were supplied ad libitum. The female mice used for all experiments were healthy, 23–26 days old, with no sign of stress or discomfort. All animal procedures and experimental protocols were approved by the Animal Welfare Committee of the Czech Academy of Sciences (Animal Ethics Number 66866/2015‐MZE‐17214, 18 December 2015).

### 2.2 Oocyte preparation

Female mice were hormonally stimulated with 5UI PMSG – Pregnant Mare's Serum Gonadotropin (Folligon, Intervet International B.V., Boxmeer, The Netherlands) at 15:00 (the eighth hour of light cycle) on the first day of the protocol. 5UI of hCG – human Chorionic Gonadotropin (CG10, Sigma‐Aldrich, St. Louis, MI, USA) were applied to mice at 13:00 on the third day of the protocol (46th hour after using PGMS). After 12 h, the female mice started ovulating. At 09:00 on the fourth day of the protocol, female mice were sacrificed by cervical dislocation and both ampullas of fallopian tube were isolated and placed in preheated M2 medium (M7167, Sigma‐Aldrich®, St. Louis, MI, USA). Cumulus‐oocytes complex (COC) was released into the M2 medium by ampulla tearing. In the next step, for releasing cumulus cells, COC was transferred into a fresh 100 µL drop of M2 medium with hyaluronidase (concentration 0.1 mg/mL) (hyase, from bovine testes, H4272, Sigma‐Aldrich®), covered with high viscous paraffin oil (P14501, Carl Roth, Germany) and left in the incubator (set on 37°C, 5% CO_2_) for 10 min.

After washing in M2 medium, the cumulus‐free eggs were transferred into a drop of Tyrode's solution (T1788 Sigma‐Aldrich®) to remove *zona pellucida*. The zona‐free eggs were washed 2× in 1 % BSA in PBS and fixed with 3.7 % paraformaldehyde (P6184, Sigma‐Aldrich®) for 20 min and washed 2× in 1% BSA in PBS. The oocytes were incubated overnight in 4°C in a drop of primary antibodies rat monoclonal anti‐CD9 (KMC8.8) (sc18869, Santa Cruz Biotechnology, Inc., Dallas, TX, USA) diluted 1:50 in 1% BSA in PBS and rabbit polyclonal anti‐Folate receptor 4 (Juno) (abx102438, Abbexa, UK) diluted 1:50 in 1% BSA in PBS, followed by 1 h incubation with secondary antibodies anti‐rat IgG Abberior STAR 635P (Abberior GmbH, Germany) and anti‐rabbit IgG Abberior STAR 580 diluted 1:100 in 1% BSA in PBS at room temperature (RT) and washed 2× in 1% BSA in PBS.

### 2.3 Adhesion of 80 nm gold beads to the oocyte surface

Gold beads with a volume of 500 µL of stock solution (Gold nanoparticles, 80 nm diameter, OD 1, stabilised suspension in 0.1 mM PBS, 753661 Sigma‐Aldrich®) were pelleted (RT, 10,000 × *g*) and re‐suspended in 500 µL 1× PBS buffer. Immunostained oocytes were transferred into the gold beads suspension before the mounting procedure. The oocytes were incubated 20 min at RT with gentle agitation. After the incubation, the oocytes were washed 3× in PBS and processed according to the *extended protocol* mounting procedure.

### 2.4 Oocyte sample preparation and mounting finalisation

The oocyte samples were prepared using either the standard protocol or the extended protocol, with the sample mounting process being identical for both procedures.

#### 2.4.1 Standard protocol

The samples were mounted according to the standard procedure for light microscopy^11^; the oocyte was transferred from the 1% BSA in PBS used for washing through a series of 5%, 20%, 50%, and 70% glycerol solutions, incubated for a minimum of 10 min at each step and finally mounted to the commercially available mounting medium Vectashield (Vector Lab., Burlingame, CA, USA).

#### 2.4.2 Extended protocol

In this procedure, gradual ethanol (EtOH) dehydration steps and gradual transfer to the mounting medium were added before final specimen mounting. The oocyte, stored in 1% BSA in PBS, was first transferred to 50% EtOH in water and incubated for 10 min at RT. Following this, the oocyte was incubated in 96% EtOH for 20 min at RT. It is important to note that the oocyte cannot be directly transferred from PBS to 96% EtOH, as the sudden mixing of solutions could damage delicate structures or even tear the specimen.

From 96% EtOH, the oocyte was transferred to 50% TDE in EtOH, incubated for 20 min at RT, and finally transferred to 100% TDE, where it was incubated for another 20 min at RT. It is important to note that directly transferring the oocyte from 96% EtOH to 100% TDE causes vigorous mixing at the phase interfaces of mixing liquids, which is highly likely to damage the oocytes. Even during the transfer to 50% TDE in EtOH, we recommend handling the specimens with care and gently mixing the solution around the specimen to prevent damage. In the next step, the oocyte was transferred to a cover glass and mounted in the TDE‐based mounting medium AD‐MOUNT C (ADVI, Říčany, CZ). At this stage, it is possible to use a 97% TDE solution in water for the mounting, as previously mentioned,^9^ or any other TDE‐based mounting medium. For observation and handling of oocytes throughout the procedure, a standard stereomicroscope was used in the initial steps. Nevertheless, because of the dehydration and transfer of oocytes to solutions with high *n_D_* in the final steps of the procedure, oocytes started to be nearly invisible in standard bright field/dark field or opaque contrast. Therefore, use of the fluorescence imaging mixed with the opaque contrast was necessary for visualisation and handling of oocytes in the final steps of the procedure.

#### 2.4.3 Final step of mounting procedure

For the final mounting of oocytes, prepared using either the *standard protocol* or *extended protocol*, the oocyte was carefully transferred into a drop of the selected mounting medium. A defined amount of mounting medium was placed in the centre of a commercially available mounting spacer, the AD‐Seal (ADVI, Ricany, CZ), with a thickness of 150 µm and an inner space diameter of 9 mm, which had been previously adhered to the slide. The use of mounting spacers (double‐sided, thickness‐defined inserts) prevents any mechanical damage to the specimen. Finally, the cover glass was gently placed over the spacer to complete the system. The adhesive surface of the spacer ensures that no additional sealing is required, as it becomes immediately and permanently fixed.

### 2.5 Confocal scanning and super‐resolution acquisition

For both classic confocal scanning and STED super‐resolution acquisition, the Leica TSC SP8 STED 3X microscope equipped with pulse white light laser (WLL2) for the excitation and pulse 775 nm laser for the emission depletion was used. Image acquisition was performed using the LAS X 3.5.6 software (Leica Microsystems, DE).

Acquisition settings for confocal imaging at Figure 2: 100× 1.4 NA STED oil objective, oil *n* = 1.518, pinhole 1AU according to the excitation wavelength 580 nm, pixel size *XY*: 175 nm × 175 nm with z‐step size 250 nm, detection on Hybrid Detector (HyD) in photon counting mode, emission captured in the interval 587–617 nm, interval for the time gating window: 0.3–10 ns.

For Figure 3, the confocal and 3D STED comparison was needed. The same pixel size was used for standard confocal and SR imaging. For the *XZ* orthogonal view acquisition, the *XZ* scan feature of the microscope was used. Confocal settings: 100× 1.4 NA STED oil objective, oil *n* = 1.518, pinhole 1AU according to the excitation wavelength 580–640 nm, pixel size *XY* or *XZ*: 19 nm × 19 nm, detection on HyD in photon counting mode, emission captured in the interval 587–617 nm or 647–728 nm, interval for the time gating window: 0.3–10 ns for both channels. In 3D STED acquisition, the pulsed 775 nm depletion laser with 60% 3D STED, compensated with additional line accumulation of incoming photons, was used. The 3D STED acquisition was performed with 0.6 AU pinhole size.

Figure 4 showing the oocyte with gold beads adhered on its surface was acquired in a combination of reflection and fluorescence settings of the acquisition mode – the visualisation of excitation (488 nm)/depletion (775 nm) PSF was performed on PMT detectors (600 V), while AOBS was in reflection mode while the counter‐visualisation of the oocyte surface (Juno protein – AlexaFluor 488) was acquired in fluorescence on HyD (photon counting mode). The visualisation of depletion 2D STED ‘doughnut‐like’ PSF was performed with 0% 3D STED, while the 3D STED PSF shape was acquired with 100% 3D STED option.

Figure 5 was acquired with the accent on standardised comparison of highest resolution reached on the oocyte surface on ‘close’ and ‘far’ from the coverslip region, proximal and distal regions. Acquisition in 2D STED imaging mode on 100× 1.4 NA STED oil objective, oil *n* = 1.518, pinhole 0.6AU according to the excitation 640 nm, pulsed 775 nm depletion laser with 0% 3D STED, pixel size *XY*: 14 nm × 14 nm, one‐plane image, detection on HyD in photon counting mode, emission interval 647–728 nm, interval for the time gating window: 0.3–10 ns. For the FRC resolution calculation method, it is important to obtain two images of the same layer that differ in the noise. Therefore, to minimise differences between the images, we performed the acquisition in two identical between‐lines sequences.

Figure 6 was acquired as a z‐stack in 3D STED imaging mode, with settings: 100× 1.4 NA STED oil objective, oil *n* = 1.518, pinhole 0.6AU according to the excitation wavelength 580 nm or 640 nm, pulsed 775 nm depletion laser with 60% 3D STED, compensated with additional line accumulation of incoming photons, pixel size *XY*: 22 nm × 22 nm and z‐step size 94 nm, detection on HyD in photon counting mode, emission interval 587–617 nm or 647–728 nm, interval for the time gating window: 0.3–10 ns for both channels. While the calculation of the resolution was performed on raw images, for the visualisation we applied gentle deconvolution to reduce the noise.

### 2.6 Calculation of image resolution

The computed resolution depends on the signal‐to‐noise ratio, spectral image content and effective optical transfer function. Thus, it is more bias‐independent compared to the more commonly used selective FWHM measurement. In principle, it is more suitable for isotropic image data; the FRC method for the measurement of the lateral resolution in XY optical sections was used,^22^ its implementation on the Fiji – ImageJ open‐source software platform (https://imagej.net/Fiji).^15^ The FRC plugin is available from the PTBIOP Update Site. It requires two images of the same scene to be loaded that differ only in noise content. Subsequently, the FRC curve is calculated, and a fixed threshold value of 1/7 was used in our case to estimate the resolution.

### 2.7 Software for image finalisation and visualisation

The image sections, maximal projections and orthogonal views were prepared on the Fiji – ImageJ open‐source software platform (https://imagej.net/Fiji).^15^ The raw super‐resolution (SR) images of the whole oocyte were processed, using the STED deconvolution module in Huygens Professional software (Scientific Volume Imaging, NL; version 20.10). The SNR for 3D STED images of the oocyte was estimated around value 7 and 5 for 580 and 640 nm excitations channels, respectively; the number of deconvolution iterations automatically stopped on threshold value 0.05; the CMLM method was used. For the volume reconstruction, 3D visualisation and movie creation, Imaris software (Bitplane AG, CH; version 9.6) was used.

## 3 RESULTS

Our previous study^16^ investigated the spatial relationship between two key oocyte surface proteins, Juno^17^ and CD9^18^, ^19^, ^20^ within oocyte plasma membrane. We aimed to visualise their precise localisation within different compartments of the membrane at nanoscale resolution to address questions about their potential interaction within the same protein network. However, the conventional confocal scanning microscope is not able to resolve given structures in sufficient resolution. Therefore, we aimed to use 3D STED for oocyte acquisition.

The standard sample preparation, which involves gradually transferring the oocyte from the water‐based staining solution to the mounting medium through a series of increasing glycerol concentrations and final mounting in Vectashield,^11^ results in pronounced spherical aberration. This aberration compromises the use of 3D STED for in‐depth imaging due to its sensitivity to the co‐alignment of excitation and depletion lasers. To address this issue, we introduced several steps in the finalisation and mounting procedure that effectively dehydrate the specimen and transfer it to the TDE‐based mounting medium. As a mounting medium with a TDE‐based composition declared by the producer, we used the commercially available AD‐MOUNT C, which has a refractive index equal to that of glass. This methodological approach, detailed in this paper, was crucial in achieving the high‐resolution images essential for our study on Juno and CD9.^16^ For the following method description, we refer to the originally published oocyte sample preparation procedure^11^ as the ‘*standard protocol*’ and the new sample finalisation approach presented in this study as the ‘*extended protocol’*.

### 3.1 Extended protocol implementation

To prepare oocytes for 3D STED microscopy, we focused on two main aspects: dehydrating the specimen and transferring it to the mounting medium. The first step involved thoroughly removing water from the sample through alcohol dehydration. The standard gradual transfer from a water‐based buffer to a high RI mounting medium is complicated by the high viscosity of these chemicals, which can prevent complete water removal. Since air‐drying is not possible due to the fragility of the oocytes, which would collapse, we introduced a gentle alcohol dehydration step. This dehydration process was initiated immediately after the final staining step in 1% BSA in PBS. The procedure involved transferring the oocytes to the 50% ethanol in water, followed by a transfer to 96% ethanol, with a 20min incubation at each step to allow for equilibration. After dehydration, the specimen was gradually transferred from 50% TDE in ethanol to 100% TDE. Finally, the sample was mounted in AD‐MOUNT C, a commercially available mounting medium with a refractive index resembling glass (RI 1.518).

TDE is a syrupy, colourless, nontoxic liquid that is miscible with both water and ethanol, as used in this procedure. Its use for final mounting is advantageous, not only because of its effectiveness as a high RI mounting solution^9^ but also because of its ability to act as a rapid optical clearing agent for thick biological structures.^21^ The combination of these steps ensures a consistent PSF shape for the excitation and depletion lasers throughout the entire sample volume, enabling the acquisition of homogeneous 3D STED high‐resolution images of thick samples.

**FIGURE 1:**
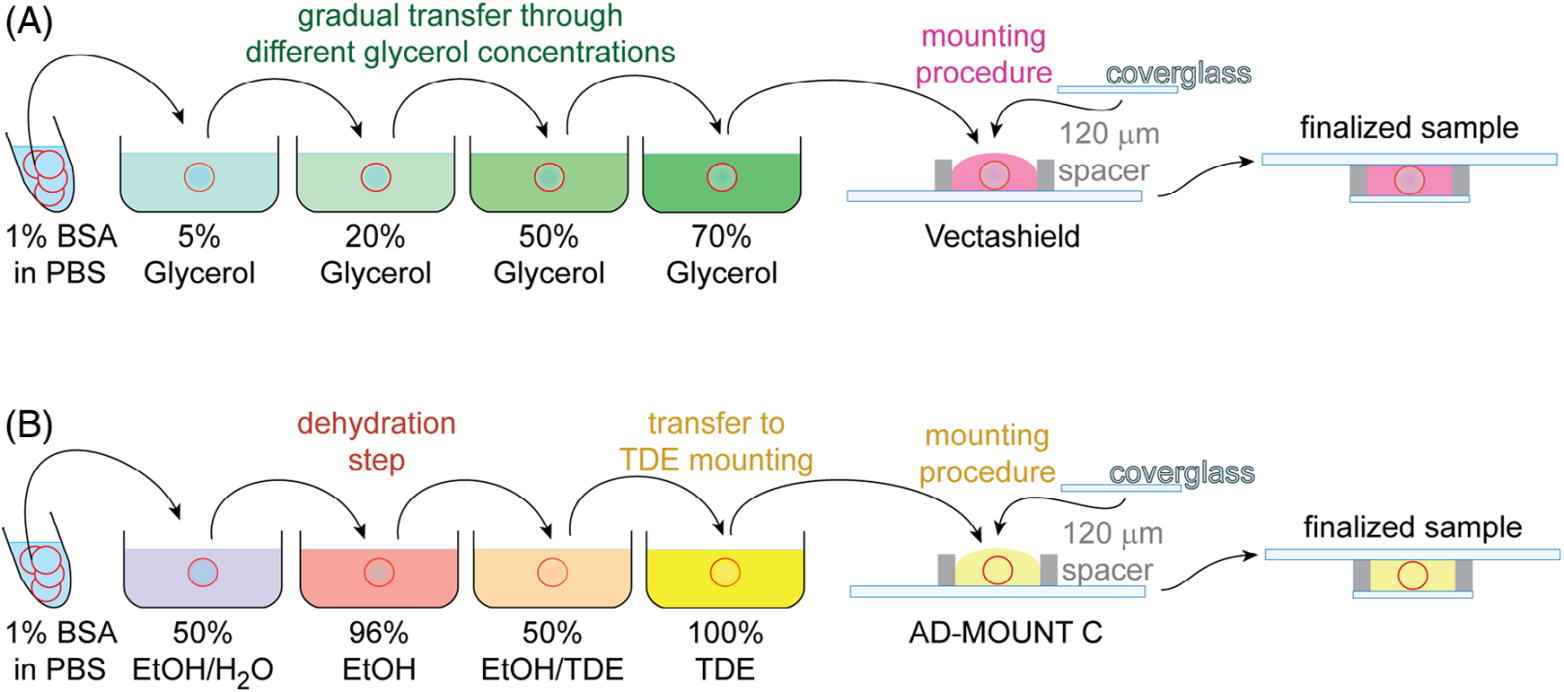
**Schematic representation of the final mounting workflow for the *standard protocol* and e*xtended protocol***. (**A)** *Standard protocol*: The sample (represented by the red circle) is transferred from a water‐based buffer through a series of glycerol solutions, starting at 5% and increasing to 70%, with a 10 min incubation at each step to equilibrate the gradient between the glycerol solution and the sample's surrounding environment. The process is finalised by mounting the sample in Vectashield mounting medium (Vector Laboratories, Burlingame, CA, USA). (**B)** *Extended protocol*: The sample is first transferred from the water‐based buffer to a 50% ethanol solution, followed by 96% ethanol for dehydration. The sample is then moved through a 50% TDE in ethanol mixture to 100% TDE, with a 20min incubation at each step. Finally, the sample is mounted in AD‐MOUNT C mounting medium (ADVI, Ricany, CZ).

As a parallel control procedure, we used the *standard protocol* that involves gradually transferring the sample from a water‐based staining solution to the mounting medium through a series of increasing glycerol concentrations^11^ at 5%, 20%, 50%, and 70% glycerol in PBS, with 10 min incubations at each step to equilibrate the gradient between the glycerol solution and the sample environment. This process was finalised by mounting to the Vectashield mounting medium (Figure 1A). We compared this procedure with our newly implemented *extended protocol* (Figure 1B).

### 3.2 Extended protocol preserves the signal intensities within entire specimen

To verify the suitability of the extended protocol for 3D STED imaging, we compared the optical properties of samples prepared using both the *standard protocol* and *extended protocol*. For oocyte surface visualisation, we indirectly immunolabelled the Juno protein^17^ on the oocyte surface. After the staining procedure, we divided the oocytes into two experimental groups. One group was prepared according to the *standard protocol*, while the other followed the *extended protocol*. Although the primary goal was to assess the performance of the samples in 3D STED imaging, we initially used conventional confocal microscopy for preliminary comparisons. After acquiring the oocyte images, we evaluated the image quality across the entire sample volume.

To assess optical homogeneity and transparency, we compared the signal intensities of the acquired structures along the *z*‐axis by analysing the point spread function (PSF) shape on opposite sides of the oocytes. Typically, optical quality decreases with increasing sample depth. For precise comparison, we defined two orientations of the oocyte relative to the microscope objective: ‘top’ and ‘bottom’. The ‘bottom’ refers to the part of the oocyte closest to the microscope objective and the coverslip, while the ‘top’ refers to the side furthest from the coverslip. We used these terms solely to describe the oocyte's orientation during imaging and not to imply any intrinsic properties of the oocyte.

**FIGURE 2:**
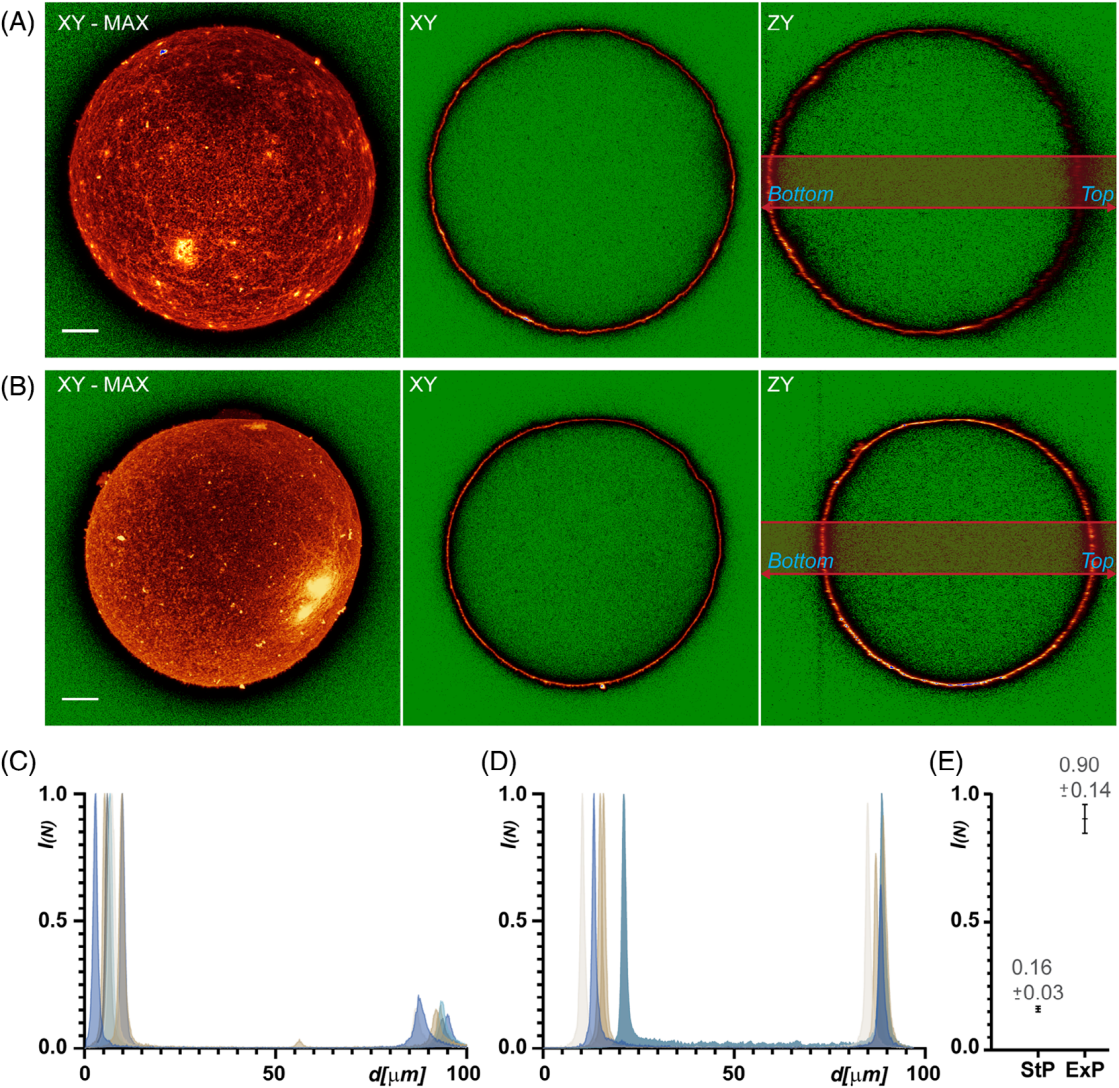
**Comparison of signal intensity differences and fading in deeper layers, as demonstrated in confocal images of oocytes prepared using the *standard protocol* and the *extended protocol***. The deeper part of the object refers to layers situated farthest from the objective, represented on the right side of the *ZY* orthogonal views, labelled as the ‘top’. The regions of the oocyte closest to the objective are labelled as the ‘bottom’. (**A, B)** Representative confocal sections in *XY* and *ZY* (orthogonal) layers, along with *XY* maximum projection images, of oocytes prepared using the (**A**) *standard protocol* and the (**B**) *extended protocol*. The ‘glow’ lookup table was used to intuitively visualise changes in signal intensities: green indicates zero values; dark red, red, yellow, and white represent the dynamic range; and blue highlights maximal values (image saturation). The scale bars represent 10 µm. In the *standard protocol* (**A**), spherical aberration results in signal fading and structure smearing at the top of the object. In contrast, the *extended protocol* (**B**) demonstrates minimal spherical aberration, with the clearing effect preserving signal intensities, resulting in almost no visible fading and a fairly homogeneous structure shape in the top of the specimen. (**C, D)** Charts visualising the normalised signal intensity ‘*I*_(_ *_N_*_)_’ on the *Y*‐axis against the distance ‘*d*’ into the sample on the *X*‐axis, representing plot profiles from the *ZY* orthogonal views of confocal images. Chart (**C**) shows the plot for the *standard protocol*, while (**D**) shows the plot for the *extended protocol*. In both (**A**) and (**B**), signal intensities were measured from the area under the light red‐grey rectangle in the *YZ* orthogonal projections. Area measurements were used instead of simple line profiles to minimise noise in the plotted data. (**E)** The chart represents the average decrease in intensities ‘*I*_(_ *_N_*_)_’ in the top area, normalised to the intensities in the bottom area. The average and standard deviation are plotted, with corresponding values shown for oocytes processed using the *standard protocol* (StP) and the *extended protocol* (ExP).

The oocytes from both groups were visualised using a confocal scanning microscope to obtain z‐stack images of the entire oocyte volume. Maximum projections and *XY* and *ZY* (orthogonal) sections of the acquired z‐stacks are shown in Figures 2A and B. As expected, the oocytes prepared according to the *standard protocol* (Figure 2A) exhibited a sharp PSF in the bottom part of the specimen, but there was a noticeable loss of signal intensity, resolution and significant blurring in the deeper, top part of the specimen. In contrast, the oocytes prepared using the *extended protocol* (Figure 2B) displayed an almost uniform PSF shape in both the bottom and top parts of the oocyte. Signal intensities were well‐preserved, and image contrast remained consistent throughout the entire imaged volume. Comparing the *extended protocol* to the *standard protocol* demonstrated significantly better optical quality in the *extended protocol* group, with improvements in signal intensity, contrast, reduced blurring and more consistent PSF shape.

To better illustrate the differences in fluorescence signal intensity between the bottom and top parts of the oocytes prepared using the two protocols, signal intensities within selected rectangular areas (Figure 2A and B) were quantified and plotted on graphs (Figure 2C and D). These graphs depict signal intensities from the bottom part of the oocyte, shown on the left side of the image, to the top part, shown on the right side of the orthogonal image. The plotted intensity values were calculated from the *YZ* orthogonal projections of the volume images.

The signal intensity profile of the sample prepared with the *standard protocol* showed significant attenuation of fluorescence in the deeper layers of the oocyte (Figure 2C). In contrast, the sample prepared with the *extended protocol* maintained nearly constant signal intensity throughout the entire volume (Figure 2D). A quantitative comparison of the normalised signal intensities from bottom to top was plotted (Figure 2E), clearly illustrating the preservation of fluorescent signal across the oocyte volume when the extended protocol was used.

### 3.3 Extended protocol ensures the lasers’ PSF co‐alignment within the sample

For successful 3D STED imaging of a large biological sample, such as a mouse oocyte (typically around 80 µm in size), two key properties are required: high transparency and a relatively constant PSF shape throughout the sample's volume. These conditions ensure the proper formation of both the *XY* and Z‐doughnuts, the precise co‐alignment of the depletion laser doughnuts with the excitation PSF, and the preservation of signal intensity in the deeper parts of the sample. Inspired by previously cited approaches for visualising 3D STED alignment in volumetric samples, we prepared a realistic calibration sample capable of visualising both excitation and depletion laser PSFs.

**FIGURE 3:**
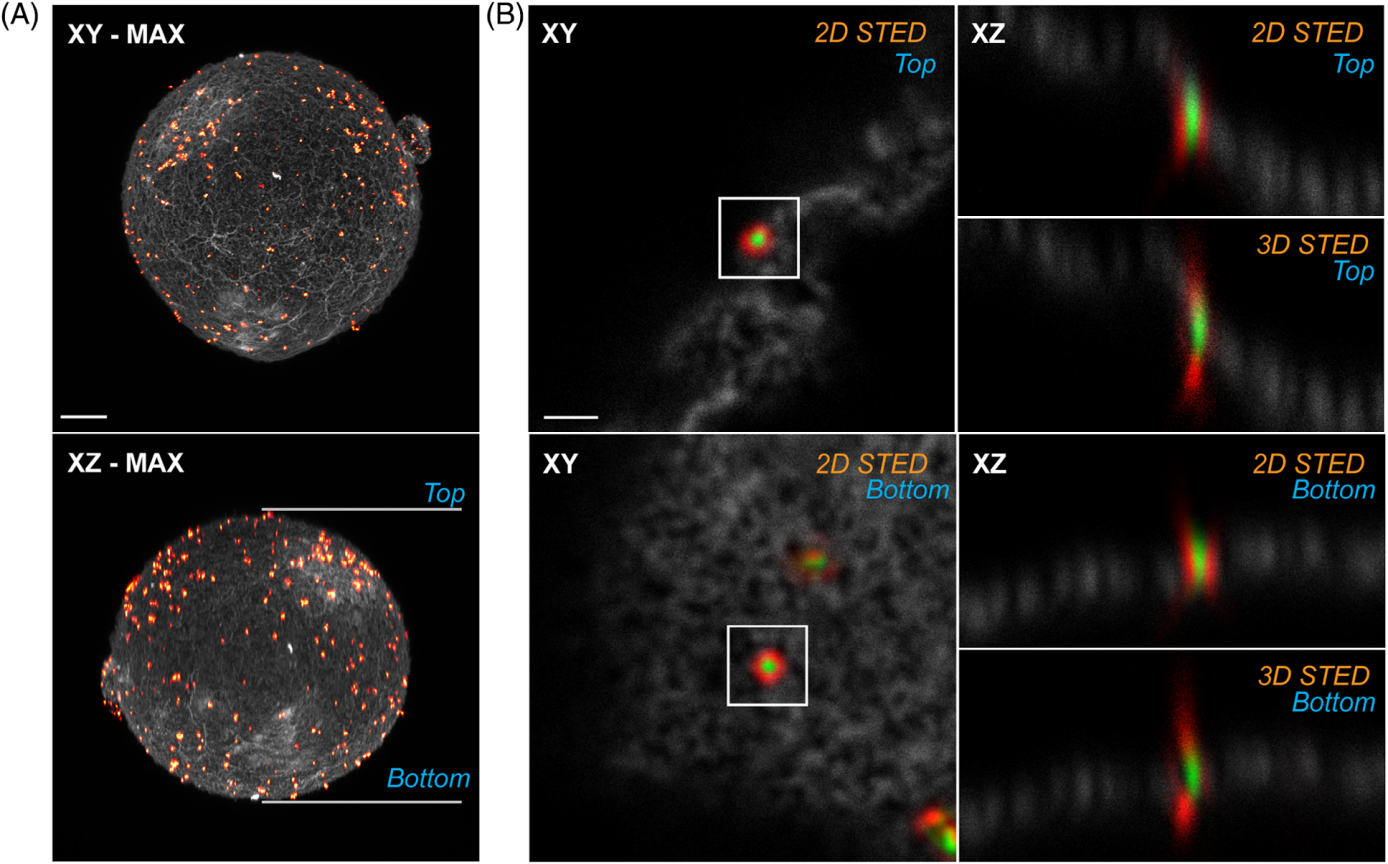
**Confocal images of an oocyte with adhered 80 nm gold beads prepared using the *extended protocol***. The images demonstrate the appropriate formation of the 2D STED and 3D STED depletion laser patterns at depths ranging from a few micrometres to approximately 80 µm. (**A)** Maximum intensity projection of the oocyte in confocal images along the *XY* and *XZ* axes. The grey channel represents the fluorescence signal of the counterstaining used to visualise the oocyte membrane, while the ‘Red Hot’ lookup table represents the visualisation of the adhered 80 nm gold beads, acquired in reflection mode. The scale bar represents 10 µm. (**B)** Confocal sections showing the oocyte membrane in the grey channel, with gold beads used to visualise the PSFs of the excitation laser (green) and the depletion laser (red). The *XY* confocal section shows the lateral co‐alignment, while the *XZ* confocal scan shows the axial co‐alignment of the excitation and depletion lasers. The positions relative to the objective are labelled as ‘bottom’ or ‘top’. The labels ‘2D STED’ and ‘3D STED’ indicate the settings of the microscope STED module, with the corresponding depletion laser PSF shape formed on the gold beads. The scale bar represents 1 µm.

To visualise the oocyte surface, we immunostained the Juno protein with AlexaFluor 488. Following the staining procedure, we adhered 80 nm gold beads directly to the oocyte surface. The oocyte sample was then processed using the extended protocol. Visualisation of the oocyte was performed in standard confocal fluorescence mode, while the adhered gold beads were visualised in confocal reflection mode (Figure 3A). The results showed that the PSF shapes of a single bead located both at the bottom and the top of the oocyte were very similar, for both the excitation and depletion lasers, in both 2D and 3D STED modes (Figure 3B). This indicates that the sensitive co‐alignment of excitation and depletion lasers is preserved throughout the entire specimen volume, suggesting the capability of 3D STED imaging across the full depth of the sample prepared using the extended protocol.

### 3.4 3D STED applicable throughout the entire specimen after extended protocol sample preparation

**FIGURE 4:**
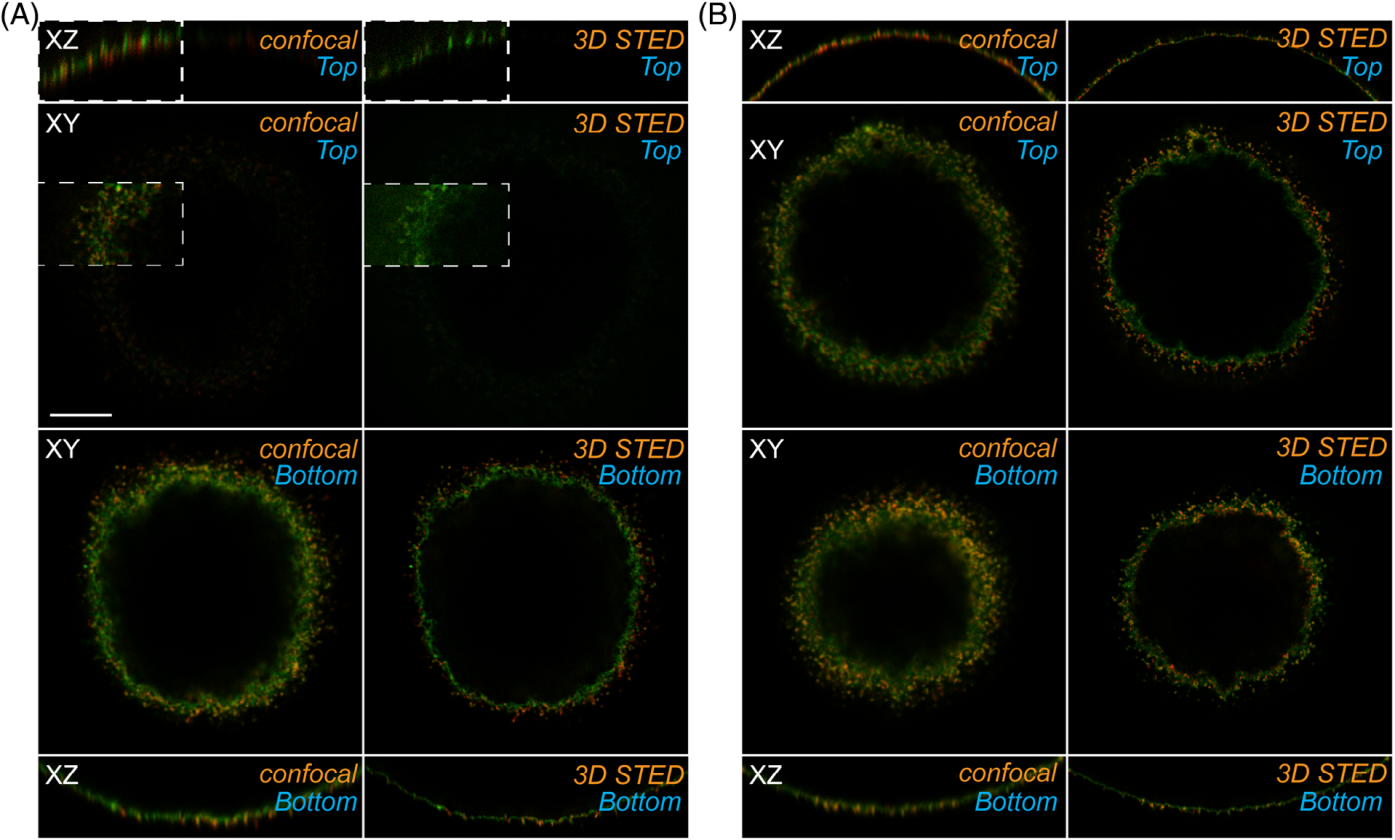
**Confocal and 3D STED images of oocytes prepared using the s*tandard protocol* and the *extended protocol***. The figure shows a sequence of images for each protocol: The first row presents a simple *XZ* orthogonal scan, while the second row shows a single *XY* section at the top of the oocyte. The third row visualises a single *XY* section at the bottom of the oocyte, and the fourth row displays a simple *XZ* orthogonal scan of the bottom region. The method used for image acquisition is labelled as either ‘confocal’ or ‘3D STED’. The scale bar represents 10 µm. (**A)** The *standard protocol* yields images with standard confocal quality at the bottom, suitable for 3D STED super‐resolution imaging. However, at the top region, the signal intensity is weak and fades, resulting in unsuccessful 3D STED imaging. To highlight the weak signals and demonstrate this issue, we nonsystematically increased the intensities of the images within the white‐bordered squares located at the top. (**B)** The *extended protocol* produces confocal and 3D STED super‐resolution images, with fully comparable quality at both the bottom and top, with no significant fading of signal intensities.

To verify the applicability of the extended protocol for 3D STED imaging, we used oocytes with immunolabelled membrane proteins Juno^17^ and CD9,^18^, ^19^, ^20^ which are important for our research. The immunolabelled oocytes were processed, using both the *standard protocol* and *extended protocol*. A comparison of standard confocal images and 3D STED images obtained using a 60% z‐doughnut is shown in Figure 4. The application of 3D STED significantly increased both lateral and axial resolution at the bottom of the specimen, regardless of the protocol used. However, significant differences were observed at the top of the specimen (Figure 4A and B).

Specimens prepared using the *standard protocol* exhibited a dramatic loss of signal intensity and smearing of signal localisation in the structures at the top of the specimen (Figure 4A). This preparation method proved ineffective for imaging the entire oocyte using 3D STED. In contrast, oocytes processed with the *extended protocol* showed excellent preservation of signal intensity and resolution in both the bottom and top regions, with structures resolved comparably throughout the entire oocyte volume (Figure 4B). These results demonstrate that the extended protocol is a suitable sample preparation technique for super‐resolution imaging of large biological structures, enabling the visualisation of the entire oocyte volume using 3D STED microscopy.

### 3.5 Comparable resolution within entire specimen after extended protocol sample preparation

**FIGURE 5:**
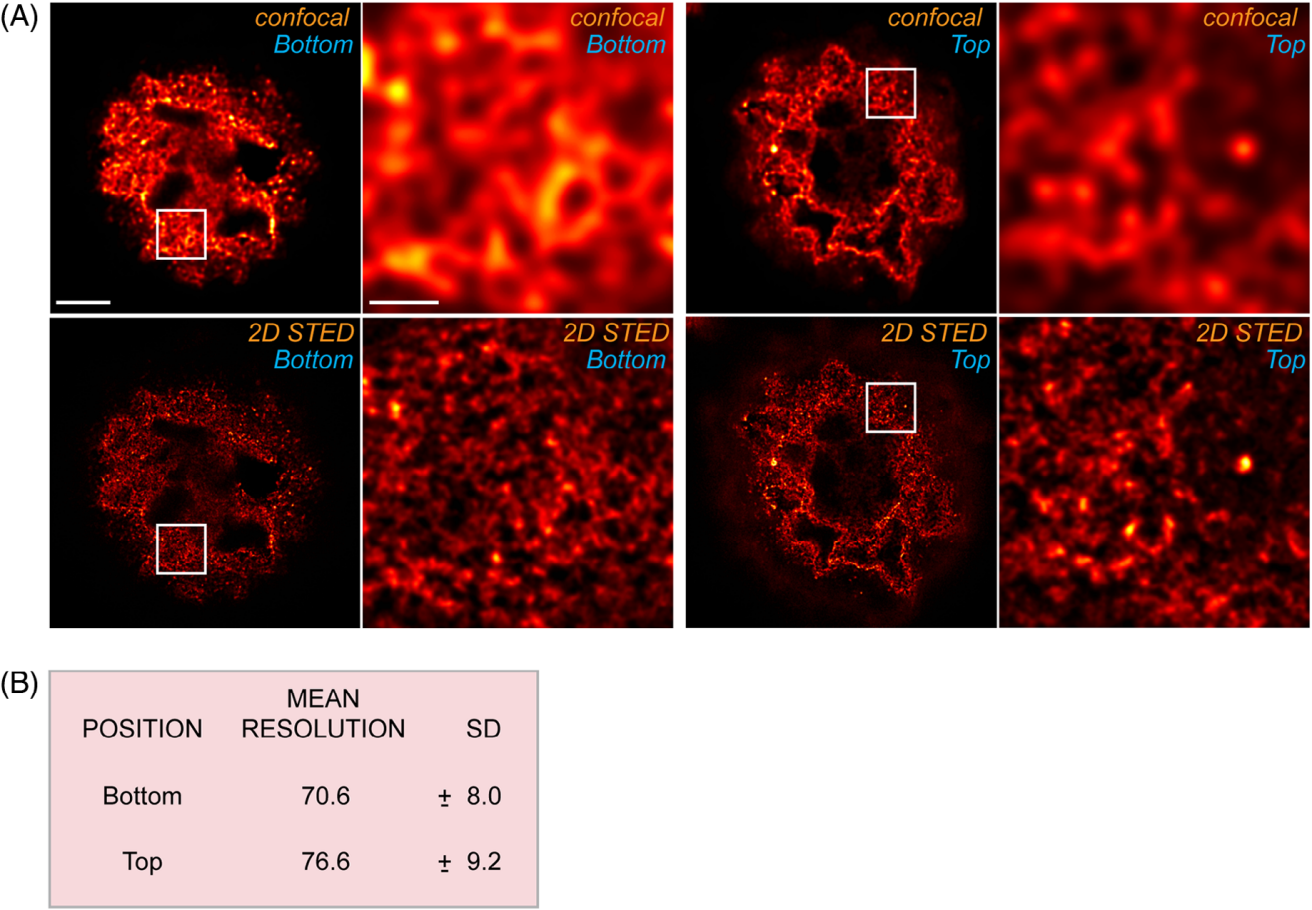
**The image resolution achieved with 2D STED approach within the oocyte sample**. (**A**) Comparison between the confocal (labelled as ‘confocal’) and 2D STED (labelled as ‘2D STED’) acquisition methods at the bottom and top positions of the oocyte. The scale bar represents 10 µm in the overview image and 1 µm in the zoomed‐in area. (**B**) A table quantifying the mean and standard deviation of the resolution, as calculated by the FRC method, for images acquired from the bottom and top regions of the specimen.

To evaluate how much the resolution varies between the bottom and top regions of the specimen prepared using the *extended protocol*, we measured the resolution using the Fourier Ring Correlation (FRC) computation method.^22^ Optical sections were taken from both the bottom and top areas of the oocyte for this analysis. FRC measurements were conducted on raw data, and the images were conservatively deconvolved to reduce noise for clearer visualisation (Figure 5A). To ensure robust resolution measurements, we analysed image data from five independently prepared oocytes. The results indicated that the resolution achieved by 2D STED microscopy was comparable between the bottom (70.6 ± 8.0 nm) and top (76.6 ± 9.2 nm) regions (Figure 5B).

### 3.6 3D STED acquisition of entire oocyte enabled by extended protocol sample preparation

**FIGURE 6:**
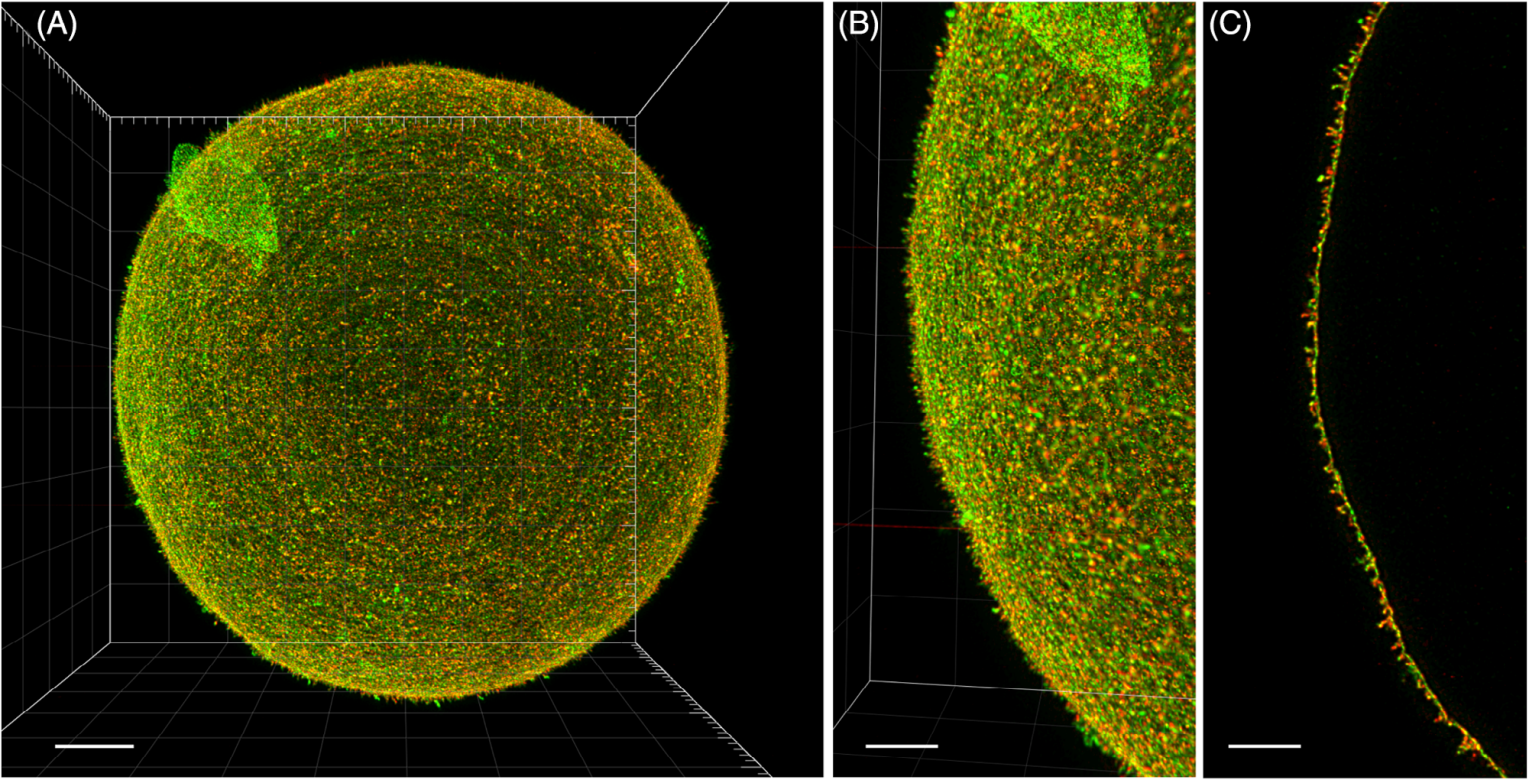
**The visualisation of the final deconvolved two‐channel 3D STED super‐resolution volume reconstructed image of the entire mouse oocyte**. This figure provides an overview of the oocyte and reveals detailed microvillar structures visualised with immunofluorescent staining of Juno (green) and co‐localised CD9 protein (red) in the zoomed‐in area. (**A**) 3D maximum projection of the full z‐stack of the entire oocyte using 3D STED microscopy, and (**B**) a close‐up of a selected area from the entire oocyte image. The scale bars represent (**A**) 10 µm and (**B**) 5 µm. (**C)** A representative maximum intensity projection of 10 selected optical sections around the equatorial layer of the oocyte in the super‐resolution 3D STED image. The scale bar represents 5 µm.

For complete acquisition using two‐channel 3D STED microscopy, the oocyte sample was prepared according to the *extended protocol*. After acquisition, the raw images were deconvolved using the STED option in Huygens Professional software. This deconvolution process maximises the recovery of acquired structures, improves the signal‐to‐noise ratio, and restores super‐resolution image quality. The final images clearly reveal the distribution details of both Juno and CD9 proteins across the entire oocyte surface. This technique allows for precise signal localisation at nanoscale resolution, with consistently high resolution throughout the entire specimen (Figure 6A and Video S1). The resulting super‐resolution images reveal extremely fine microvillar structures (Figure 6B) that, until now, have only been visualised by electron microscopy.^23^ Individual oolemma compartments, such as the microvillar membrane and the planar membrane located between *microvilli*, are clearly distinguishable. We were able to demonstrate^16^ the distinct localisation of these two proteins within the individual oolemma compartments (Figure 6C).

## 4 DISCUSSION

Super‐resolution (SR) techniques in light microscopy have significantly advanced our ability to visualise fine biological structures and analyse their composition at nanoscale resolution. Despite their broad applicability, SR methods face limitations imposed by the thickness of the sample and the depth of the region to be imaged. The confocal‐based method, STED, naturally removes out‐of‐focus light, enabling the acquisition of deeper layers within the sample.^24^ The 3D STED approach further enhances resolution in both lateral and axial dimensions.^25^ However, its effectiveness is still constrained by the sample thickness. In 3D STED, the precise overlap of the excitation and depletion laser PSF patterns is crucial. In deeper layers of the sample, the geometry of the excitation and depletion beams is affected differently, which can impair the method effectiveness.^26^

Spherical aberration, a key factor limiting image quality in thick samples, can be achieved using a dedicated objective with an appropriate immersion medium, such as a glycerol or water immersion objective with correction collar, which match the refractive index (RI) of the mounting medium used for sample finalisation.^27^ However, when maximal resolution is required, a high NA oil immersion objective is preferred. Using high NA oil objectives requires improvement of the sample optical quality by increasing the RI of the sample environment to match the RI of glass – the material of both the objective and the cover glass.

Our study presents a significant advancement in overcoming these limitations through an optimised sample preparation method. This method, referred to as the *extended protocol*, focuses on achieving optical homogeneity by implementing two critical steps: thorough water removal and appropriate mounting using a glass‐equal RI medium. The removal of water from the sample in the previous *standard procedure* for oocyte preparation for fluorescence microscopy involved gradually replacing water with increasing concentrations of glycerol, followed by final assembly in Vectashield (or a similar RI mounting medium).^11^ This method is suitable for robust confocal microscopy, particularly when combined with techniques to compensate for light attenuation at increasing image depths.^28^ However, it is insufficient for the more sensitive 3D STED method, where spherical aberration cannot be adequately compensated, and the fundamental principle of the method is compromised. In contrast, our *extended protocol* employs alcohol‐based dehydration to effectively remove residual water, preventing structural collapse of the fragile oocyte during preparation.

A crucial aspect of this protocol is the transition of the sample through an ethanol‐TDE solution into a mounting medium that is fully compatible with TDE. For the final mounting, it is possible to use a 97% TDE solution in water^9^ or select another TDE‐based mounting medium. This TDE compatibility is essential for avoiding unwanted optical issues that could arise from incompatible mixtures causing internal phase transitions. In our study, we used AD‐MOUNT C, a commercially available mounting medium with a glass‐like refractive index (RI) of 1.518, with its TDE‐based composition as declared by the producer. One of the key properties of TDE is its ability to tune the RI to match that of glass.^9^ This step is crucial for minimising spherical aberration and maintaining a uniform PSF shape throughout the sample depth, which is particularly relevant for oocytes with a thickness of approximately 80 µm. Matching the RI to that of glass is essential for achieving optimal performance with a high NA oil objective dedicated for STED acquisition. Additionally, TDE offers a rapid clearing effect,^21^ which reduces light scattering in thick samples, thereby enhancing the transparency of biological structures. As a result, the fluorescent signal is uniformly preserved across the sample's depth during excitation, depletion and emission.

However, the use of TDE is not without its limitations. One significant limitation is that, unlike the fluorescence of mRFP, the fluorescence of EGFP is quenched at TDE concentrations greater than 80%.^9^ This quenching effect limits the applicability of TDE for studies involving EGFP and highlights a need for further investigation into the fluorescence properties of EGFP under these conditions. In addition, there is an ongoing discussion within the field of microscopy about TDE's quenching effect on silicon rhodamine (SiR) dyes, which may also limit its use in specific applications. These factors should be carefully considered when selecting TDE or TDE‐based mounting media.

Given these considerations, it is also essential to optimise the incubation periods during the dehydration and TDE steps according to the specific characteristics of the sample. For instance, while our protocol was optimised for oocytes around 80 µm in size, different biological samples may require adjustments. For example, shorter incubation times were sufficient when applying this protocol to a simple adherent cell culture monolayer. Conversely, for a 100 µm‐sized organoid, much longer incubation times – lasting several hours – were necessary; data not shown. This extended duration is related to the higher structural density of the organoid. We emphasise that careful optimisation of this step is crucial for the specific type of sample being used.

The removal of water significantly improves the optical quality of the sample, while the dehydration process, conducted without drying, ensures the integrity of the sample structures and preserves the shape of the object. Mounting the sample in a high refractive index medium with a clearing effect creates an optically homogeneous and transparent environment. This approach makes the technique suitable for high‐resolution and super‐resolution microscopy methods. We successfully achieved two‐channel 3D STED imaging throughout the entire oocyte volume with consistent resolution. This achievement is particularly interesting, as oocytes are widely used as a model for studying membrane proteins, such as auxin hormone transporters.^29^ More broadly, there is a growing trend in science to move from 2D adherent cell cultures to more realistic models like organoids.^30^ We believe that this simple technique has the potential for a much wider range of applications in the field of fluorescence microscopy, including other SR techniques and various types of thicker biological specimens.

## 5 CONCLUSIONS

In this study, we present the first two‐channel 3D STED imaging of the entire volume of an oocyte, offering detailed visualisation of membrane proteins at nanoscale resolution. Our extended sample preparation protocol includes alcohol‐based dehydration and mounting in a high refractive index TDE‐based medium comparable with a refractive index of glass. This innovation successfully overcomes the limitations of previous methods that were inadequate for super‐resolution microscopy of large and fragile biological specimens such as oocytes. The co‐distribution of captured proteins within microvillar surface structures across the entire oocyte demonstrates the usability of the protocol to maintain high image quality and consistent resolution throughout the specimen's depth.

The results of this study provide solid evidence that the presented *extended protocol* is a robust and effective solution for 3D STED imaging of large biological samples. By minimising spherical aberration and preserving structural integrity, this method allows for high‐resolution imaging across the full volume of the sample. This protocol represents a promising tool for further applications such as organoids and complex tissue samples, allowing for further optimising towards enhancement of its versatility in super‐resolution microscopy.

## AUTHOR CONTRIBUTIONS

M.F. prepared all the biological samples for the 3D STED super‐resolution microscopy, collaborated in optimisation of the *extended protocol*, 3D STED imaging and image processing, and contributed to the writing of the manuscript. M.C., M.B. and H.C. contributed to image processing and manuscript writing. V.K. contributed to the method development and manuscript writing. E.V. contributed to the experimental part of the study. M.G. contributed to manuscript writing. K.K. contributed to writing the manuscript and provided funding. O.H. contributed to manuscript writing and provided funding. I.N. developed and optimised the *extended protocol* for oocyte preparation, performed the 3D STED acquisition settings and collaborated in 3D STED imaging and image processing, contributed to writing the manuscript and provided the funding.

## CONFLICT OF INTEREST STATEMENT

The authors declare no conflicts of interest.

## Supporting information



Supporting Information
